# IgE Recognition Patterns of Profilin, PR-10, and Tropomyosin Panallergens Tested in 3,113 Allergic Patients by Allergen Microarray-Based Technology

**DOI:** 10.1371/journal.pone.0024912

**Published:** 2011-09-15

**Authors:** Enrico Scala, Claudia Alessandri, Paola Palazzo, Debora Pomponi, Marina Liso, Maria Livia Bernardi, Rosetta Ferrara, Danila Zennaro, Mario Santoro, Chiara Rasi, Adriano Mari

**Affiliations:** Center for Molecular Allergology, IDI-IRCCS, Rome, Italy; Albany Medical College, United States of America

## Abstract

**Background:**

IgE recognition of panallergens having highly conserved sequence regions, structure, and function and shared by inhalant and food allergen sources is often observed.

**Methods:**

We evaluated the IgE recognition profile of profilins (Bet v 2, Cyn d 12, Hel a 2, Hev b 8, Mer a 1, Ole e 2, Par j 3, Phl p 12, Pho d 2), PR-10 proteins (Aln g 1, Api g 1, Bet v 1.0101, Bet v 1.0401, Cor a 1, Dau c 1 and Mal d 1.0108) and tropomyosins (Ani s 3, Der p 10, Hel as 1, Pen i 1, Pen m 1, Per a 7) using the Immuno-Solid phase Allergen Chip (ISAC) microarray system. The three panallergen groups were well represented among the allergenic molecules immobilized on the ISAC. Moreover, they are distributed in several taxonomical allergenic sources, either close or distant, and have a route of exposure being either inhalation or ingestion.

**Results:**

3,113 individuals (49.9% female) were selected on the basis of their reactivity to profilins, PR-10 or tropomyosins. 1,521 (48.8%) patients were reactive to profilins (77.6% Mer a 1 IgE^+^), 1,420 (45.6%) to PR-10 (92.5% Bet v 1 IgE^+^) and 632 (20.3%) to tropomyosins (68% Der p 10 IgE^+^). A significant direct relationship between different representative molecules within each group of panallergens was found. 2,688 patients (86.4%) recognized only one out of the three distinct groups of molecules as confirmed also by hierarchical clustering analysis.

**Conclusions:**

Unless exposed to most of the allergens in the same or related allergenic sources, a preferential IgE response to distinct panallergens has been recorded. Allergen microarray IgE testing increases our knowledge of the IgE immune response and related epidemiological features within and between homologous molecules better describing the patients' immunological phenotypes.

## Introduction

The prevalence of allergic diseases has dramatically increased in last decades, mostly in developed countries [1], [2]. Comprehensive and reliable allergy diagnostic testing are needed, also for therapeutic decision making [3]. In vitro diagnosis of allergic sensitizations is based on the measurement of specific IgE in sera of allergic patients [4]. Most of the currently available singleplex in vitro assays measure the IgE reactivity to extracts obtained from raw material derived from several organisms or their tissues. No information about the number of molecules involved or about the molecular profile associated with a given clinical manifestation is currently provided by extract-based testing.

A major problem is represented by the possibility that positive results, employing extracts, could be caused by IgE recognition of homologous molecules instead of molecules that represent the genuine marker of sensitization to a given biological source [5]. In the past 10 years the production of highly purified natural or recombinant allergens together with the recent advances in microarray technology provided the background for the development of a multiplexed assay as efficient means to test IgE sensitization to hundreds of allergenic molecules simultaneously in large populations [6]–[8].

Adverse reactions after the ingestion of animal- or plant-derived foods caused by IgE cross-reactive molecules shared by inhalant and food allergen sources are often seen in clinical practice [9]. These molecules are commonly defined as “pan-allergens” and represent families of homologous and structurally related proteins belonging to different biological sources (i.e. profilins, PR-10 molecules, tropomyosins) [10]–[12]. As the three molecule groups considered in the present study have different distributions in nature, namely profilins are ubiquitous eukaryotic proteins, found in both animal and plant sources, tropomyosins are highly conserved eukaryotic proteins, but are not found in plants, and PR-10 proteins are not found in animals, the “panallergen” definition has been herein used to identify allergenic molecules belonging to distant genera/orders rather than because they span across kingdoms. Profilins are actin-binding proteins having a molecular weight between 12–15 kDa, and present in the cell cytoplasm. They have been described as allergens in pollen sources and plant-derived food. Up to now 100 profilins have been described as allergenic, 55 of them in pollen from distant taxonomical species. PR-10 belong to the pathogenesis-related protein groups. They are molecules having a molecular weight of 16–18 kDa, produced in response to biotic and abiotic stresses, and described as allergens in pollen and plant-derived foods. Fifty three PR-10 have been described up to now, 17 from pollen of the *Fagales* order, whereas homologous from foods, like apple, carrot, celery, kiwi, are from quite distant taxonomical species. Tropomyosins are animal structural muscle proteins with a molecular weight of about 35 kDa. Tropomyosins are well described food allergens from crustaceans, but homologous allergens are found in arthropods not commonly used as food, like mites and cockroaches, causing allergic diseases by the inhalation route. Up to now 93 tropomyosins have been described as allergenic, 77 from shellfishes. All data on panallergens have been retrieved from Allergome (www.allergome.org), accessed April 30, 2011. This web-based platform can be used for more detailed and up to date descriptions of each allergen reported in the present study.

The aim of our study was to bring a further and more in deep insight into the distribution of the IgE reactivity to three panallergen groups (profilins, PR-10 and tropomyosins) in 3,113 individuals with a history of food allergy, rhinitis, asthma or atopic dermatitis, either looking at the reciprocal IgE positivity of each group compared to the others, or within each of the three panallergen groups. The study population is from Italy, extracted from a reported study [7], and being exposed and sensitized to the three groups of molecules. We selected these three groups of panallergens because they are well represented among the allergenic molecules immobilized on the available microarray version, and because they are distributed in several taxonomical allergenic sources, either close or distant, and having a route of exposure being either inhalation or ingestion. Thus, these three molecule groups have the highest probability to get in contact with the human immune system, though variable exposure levels could occur, largely depending on inhalant allergen seasonality, spatial (geographical) distributions, or frequency and amount of ingested food items.

## Methods

### Ethics Statement

The study was approved by the Institutional Review Board of Istituto Dermopatico dell'Immacolata – IDI-IRCCS, Rome, Italy (n. 106-CE-2005). The Institutional Review Board approved that the study is based on the oral informed consent for blood sampling obtained from patients or caregivers during the allergy consult, as IgE detection by microarray testing was part of the routine procedures in each patient diagnostic work-up. All patients, being informed by the specialist performing demographic and clinical data collection and suggesting them the IgE testing as reported in the present study, approved the study itself.

### Study population

Data herein reported have been extracted and further analyzed from the total of 23,077 subjects previously reported by us [7]. Briefly, unselected consecutive subjects, 61.2% female; age range between 1 and 98 years old, referred for a history of food allergy, rhinitis, asthma, atopic dermatitis, latex allergy, acute or chronic urticaria to the Center for Molecular Allergology (IDI-IRCCS, Rome, Italy) from March 2006 to December 2007 [7]. Demographical (age and gender) and clinical (respiratory and food ingestion-related symptoms) data were recorded for all patients at history taking by means of the InterAll software for clinical data recording (InterAll version 3.0, Allergy Data Laboratories s.c., Latina, Italy) [13].

Three thousand, one hundred and thirteen individuals (49.9% female; age range between 2 and 96 years old) were selected on the basis of their IgE reactivity detected using the Immuno-Solid phase Allergen Chip (ISAC) system (VBC-Genomics, Vienna, Austria). Candidates had to be IgE positive to at least one allergen of the following group of panallergens: profilins (Bet v 2, Cyn d 12, Hel a 2, Hev b 8, Mer a 1, Ole e 2, Par j 3, Phl p 12, Pho d 2); PR-10 (Aln g 1, Api g 1, Bet v 1.0101, Bet v 1.0401, Cor a 1, Dau c 1 and Mal d 1.0108); tropomyosins (Ani s 3, Der p 10, Hel as 1, Pen i 1, Pen m 1, Per a 7). Selected subjects thus acted as our study group.

### Purified natural and recombinant allergens

Table 1 reports the characteristics of the recombinant and natural allergenic molecules listed above and used for the IgE microarray testing in this study. All the molecules studied were recombinant allergens expressed in *Escherichia coli*, with the exception of Ole e 2, Pen m 1, Pen i 1, which were naturally purified preparations. Table 1 displays the Allergome code (Allergen ID) for each molecule. Further details on single molecule characteristics are available via the Allergome web site (www.allergome.org) [13].

**Table 1:** List, characteristics, and Allergome reference code for the three panallergen groups, ranked by prevalence of IgE reactivity among and within groups.

Panallergens				IgE Reactive Patients			
Allergen	Common name	Allergome Code	Molecule Sequence Identity°	N.	%	N.	%
**Mer a 1**	*Profilin*	476	100	1,521	48.8	1181	77.6
**Hev b 8**		397	81			1019	67.0
**Bet v 2**		127	84			986	64.8
**Ole e 2** *		490	85			977	64.2
**Hel a 2**		377	75			889	58.4
**Pho d 2**		571	80			876	57.6
**Cyn d 12**		279	76			857	56.3
**Par j 3**		510	78			688	45.2
**Phl p 12**		553	82			551	36.2
**Bet v 1**	*PR-10*	89	100	1,420	45.6	1314	92.5
**Cor a 1**		232	83			960	67.6
**Aln g 1**		7	81			741	52.2
**Mal d 1**		464	65			696	49.0
**Api g 1**		40	41			163	11.5
**Dau c 1**		287	39			59	4.2
**Der p 10**	*Tropomyosin*	311	100	632	20.3	430	68.0
**Pen i 1** *		527	≈80#			326	51.6
**Pen m 1** *		872	80			313	49.5
**Ani s 3**		37	73			313	49.5
**Hel as 1**		378	64			309	48.9
**Per a 7**		542	79			272	43.0

* Natural purified molecules, all others are *E.coli*-produced recombinants. Biochemical, immunological and clinical details on each allergen are available at www.allergome.org.

°Generated using AllergomeAligner (www.allergome.org) assuming the first ranked IgE reactive allergen as reference sequence ( = 100%).

# Complete Pen i 1 sequence is not available. From peptide fragment matching and IgE reactivity it is assumed to be close to Pen m 1.

### Proteomic Allergen Microarray IgE Testing

#### IgE microarray assay

Glass microscopy slides, each bearing four reaction sites bearing immobilized allergens, as provided by the manufacturer (VBC-Genomics), were washed for 60 min in TBS-T buffer (150 mM sodium chloride, 10 mM Tris base, and 0.5% Tween 20, pH 8.0), rinsed with deionised water, and dried. Slides were placed into a humid chamber and 20 µl of undiluted serum from each patient was applied to the reaction site. After incubation with patient's sera for 120 min at R.T., slides were rinsed and washed for 15 min in TBS-T, for 5 min in deionised water and dried. To detect bound IgE antibodies, allergen chips were incubated for 60 min at room temperature with 20 µl of an Alexa Fluor 546 fluorescence-labeled (Alexa Fluor 546 protein labeling kit, Molecular Probes, Leiden, Netherlands) anti-human IgE antibody (Pharmingen, San Diego, CA) diluted 1∶1000 in TBS-T containing 5% milk powder. Afterwards, slides were washed twice for 10 min with TBS-T, rinsed with deionised water, dried, and stored in the dark until scanning.

#### Image and data acquisition, and quantification of IgE

Images were acquired by scanning allergen chips with a ScanArray Gx Microarray Analysis System (Perkin Elmer Life and Analytical Sciences, Shelton, CT) with two different laser power settings. In the first scan, 70% of the laser power (50 db) was employed to obtain the highest possible signal intensities with acceptable background noise. In a second scanning step, laser power was reduced to 35 db in order to prevent saturated signals (65.535). Both images of each spot were analyzed using the ScanArray Express 3.0 software (PerkinElmer). Captured images saved as a TIFF file were imported into the ISAC software (VBC-Genomics) which specifically process the raw fluorescence value expressed as fluorescence index of each spot. From the three fluorescence values obtained for each allergen on triplicate spots, the mean value was calculated and used for further analysis. IgE values were expressed as kU_A_/l by interpolating the mean fluorescence value with a previously established reference curve [14]–[16]. At the end of each run data were exported to the InterAll software by a real-time connectivity, and saved in the patient's record where demographical and clinical data were saved earlier by the allergy specialist.

Though several ISAC biochips with different number of spotted allergens have been used during the study timeframe (ISAC79, ISAC81, ISAC86), we analyzed results from the 23 homologous molecules immobilized on all of them. For comparative purposes we used results available the same microarray testing for Phl p 1, Par j 2, and Der p 1, all considered genuine markers of sensitization to grass and parietaria pollen, and to house dust mite, respectively. The only route of exposure for all the selected genuine allergens is inhalation.

### Clustering analysis and Statistical evaluation

To verify the molecule IgE recognition profiles, we took advantage of the Genesis software version 1.7.2, originally developed for cluster analysis of genomics data [17]. The software has been adapted to be applied to proteomics of specific IgE detected by means of the ISAC molecule-based microarray.

Relatedness of IgE recognition profiles was tested by applying unsupervised Eisen's hierarchical cluster methods [18] to the data set, encompassing allergenic molecules across all samples and using as agglomeration rule the average linkage clustering as implemented in the Genesis software [17]. Unsupervised clustering involved the sorting of both homologous allergenic molecules and IgE recognition values. The IgE recognition tree was computed on the basis of a full data set and the distance between samples were computed by using Pearson correlation as similarity measures. Molecular allergens with a similar pattern of IgE recognition were grouped as hierarchical clusters and presented as heat-maps [7]. Each square in the heat-map represent the presence (red) or the absence (black) of IgE recognition of any given tested allergens for each tested subject. The red color intensity of every single square in the heat-map is directly associated with the measured IgE concentration. Interpretation of the heat-map generated by the software can be done either visually, where clustering IgE positive allergens tends to give more homogeneous red areas, or by taking into consideration the higher or lower level of dendrograms on allergen side of the graph.

Data collected in the InterAll database were also analyzed using the SPSS/PC + statistical package (SPSS, version 15, Chicago, IL). The levels of the variables of interest in the serum (IgE) from allergic patients were summarized showing the mean ± SD, in order to provide the most easily interpretable summary measure. However, differences between the levels of these variables were also tested using the nonparametric Mann–Whitney U-test. The degree of relationship between the quantitative variables studied was analyzed using Pearson Correlation test (r). Chi-square test has been used for prevalence comparisons. Statistical significance cut-off level has been set for p<0.01.

## Results

### Descriptive statistics

A total of 3,113 subjects (49.9% female), showing an IgE reactivity to at least one molecule belonging to profilin, PR-10, and tropomyosin allergen groups, represented our study group. The median age was 30.8±17.04 years, but male subjects were significantly younger (28.3±17.5 *vs* 30.4±16.1, Mann-Whitney Rank Sum Test p <0.0001). No major differences were recorded comparing to the median age of the entire studied population (30.8±18.3 years), whilst the gender distribution was different when compared to the original general population (55.2% female) from which our study group was extracted.

As shown in Table 1, profilin reactive individuals represented the largest group of patients, whilst tropomyosin reactive patients were the smaller one. Interestingly, PR-10 reactive individuals were prevalently female (Pearson χ^2^ test 4.8 p = 0.027), whereas the opposite was observed in the case of tropomyosin reactivity (χ^2^ test 5.36 p<0.021). A slight, not significant, prevalence of male population (51.4%) was observed in profilin reactive patients, as well. Unless profilin sequence identity were above 75%, IgE recognition ranked between 76.6% and 36.2%. A broader sequence identity was recorded within PR-10 molecules, having Dau c 1 (39%) the lowest sequence identity value. IgE prevalence was the broadest as well, ranging between 92.5% and 4.2%. Tropomyosins behaved as profilins, having the lowest sequence identity above 64%, but IgE prevalence ranged between 68% and 43%.

**Figure 1:**
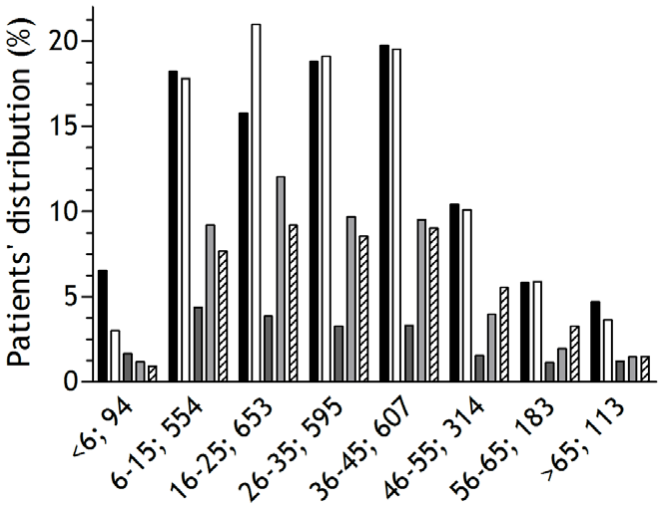
Age group distribution of the ISAC IgE panallergens-reactive population; n = 3,113. Black bars: Total 23,077 original allergic population; White Bars: Total 3,113 panallergens allergic population; Dark grey bars: PR-10; Hatched bars: Profilin; Light grey bars: Tropomyosin.

The panallergens IgE-positive cohort has been divided in eight consecutive age groups (Figure 1). Patients younger than 5 years old mainly recognized tropomyosin molecules, whilst the IgE profilin-reactive population was more prevalent between patients aged 6 to 45 years old. The prevalence of IgE reactivity to PR-10 molecules was the top ranked in patients older than 46.

**Figure 2:**
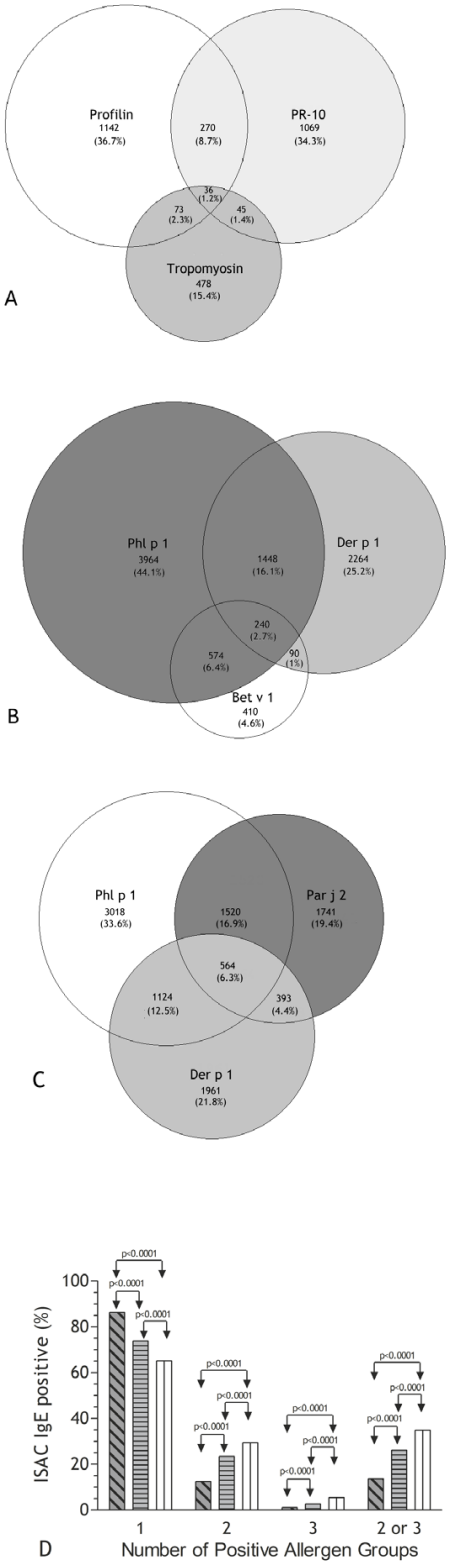
Venn diagram comparative representations of the IgE reactivity distributions. Panel A: Venn diagram of IgE reactivity distributions of the three panallergen groups, profilins, PR-10, and tropomyosin (n = 3,113); Panel B: Venn diagram of IgE reactivity distributions of genuine inhalant allergens Phl p 1, Der p 1, and including Bet v 1 when considered as a marker of genuine pollen sensitization; Panel C: Venn diagram of IgE reactivity distributions of exclusive genuine inhalant allergens Phl p 1, Der p 1, and Par j 2; Panel D: Statistical comparative evaluations of concurrent IgE reactivity to 1, 2, 3, or 2 and 3 groups in Venn diagrams shown in panel A (dark grey hatched bars), panel B (light grey horizontal line bars), panel C (white vertical line bars).

Two thousand, six hundred and eighty-eight patients (86.4%) recognized just one out of the three distinct groups of panallergens, with a preferential recognition behavior (Figure 2, Panel A). Four hundred and twenty-four (13.6%) individuals had IgE to more than one panallergen group, but, among them, only 36 individuals (1.2%) recognized molecules belonging to all the three panallergen groups (Figure 2, Panel A). Among patients having IgE recognizing more than one panallergen group, 270 individuals (8.7%) had IgE to PR-10 and profilin molecules, 73 (2.3%) to tropomyosin and profilin, and 45 (1.4%) to tropomyosin and PR-10 panallergens (Figure 2, Panel A). To verify whether such distribution was related to any bias present in the patient recruitment phase or was related to any specific allergen sensitization process, data available for genuine allergen IgE testing were analyzed as reported in Figure 2 panel B and C. Concurrent sensitization to 2 or 3 genuine allergens was recorded with a statistically significant difference when compared to the distribution recorded for panallergens.

### Bivariate Correlation within Panallergen Groups

**Figure 3:**
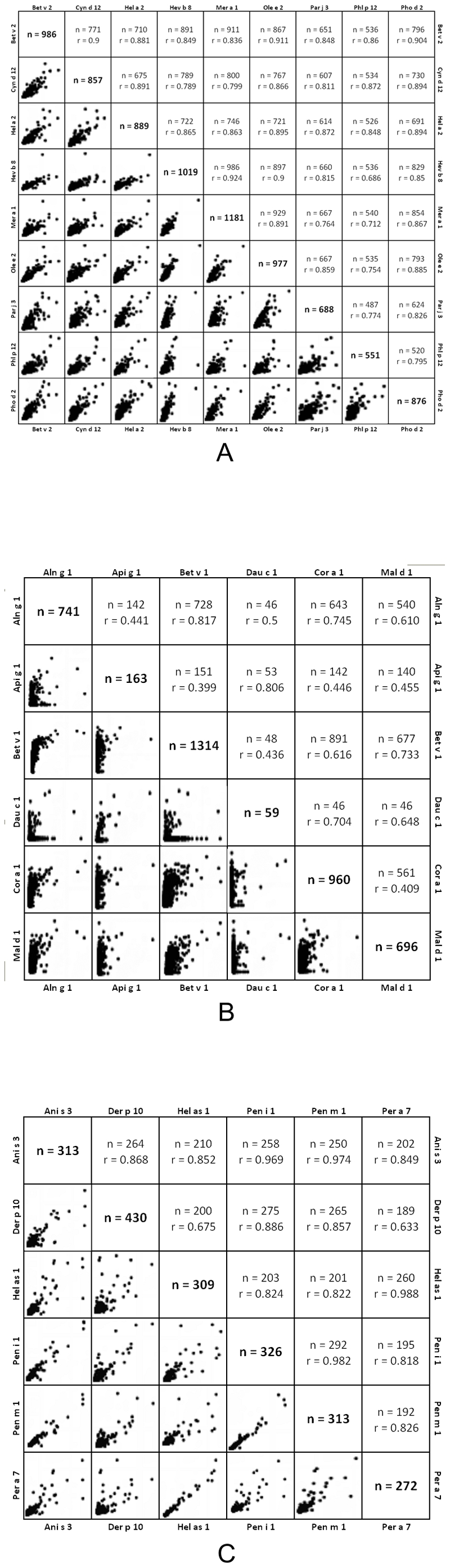
Bivariate analysis of reciprocal relationships of IgE to profilin, PR-10, and tropomyosin panallergens. The number of IgE reactive subjects and the Pearson coefficient are shown for paired allergens within the three panallergen groups. Pearson r values are reported below. Data were statistically significant in all cases (p<0.001). Panel A: Profilins; Panel B: PR-10; Panel C: Tropomyosins.

The association among the different molecules studied without distinction between independent and dependent variables was analyzed using the Pearson Correlation test. As shown in Figure 3, a significant direct relationship of IgE recognition of profilin, PR-10, and tropomyosin molecules, respectively, was recorded (p<0.001). Every single molecule belonging to a given group of panallergens, therefore, showed similar behavior of IgE recognition. In the case of PR-10 molecules, regardless the presence of a significant association between all molecules, the Pearson correlation coefficients analysis revealed a strong relationship (Pearson Correlation from 0.70 to 1) between Bet v 1, Aln g 1 and Cor a 1 from one side and between Api g 1 and Dau c 1 from the other, thus suggesting the presence of a distinct patterns of IgE recognition between PR-10 molecules belonging to inhalant and food allergens, respectively.

In the case of profilin and tropomyosin molecules, all the Pearson correlation coefficient resulted to be higher than 0.70, thus confirming a strong structural and immunological relationship between the tested molecules.

### Cluster analysis

**Figure 4:**
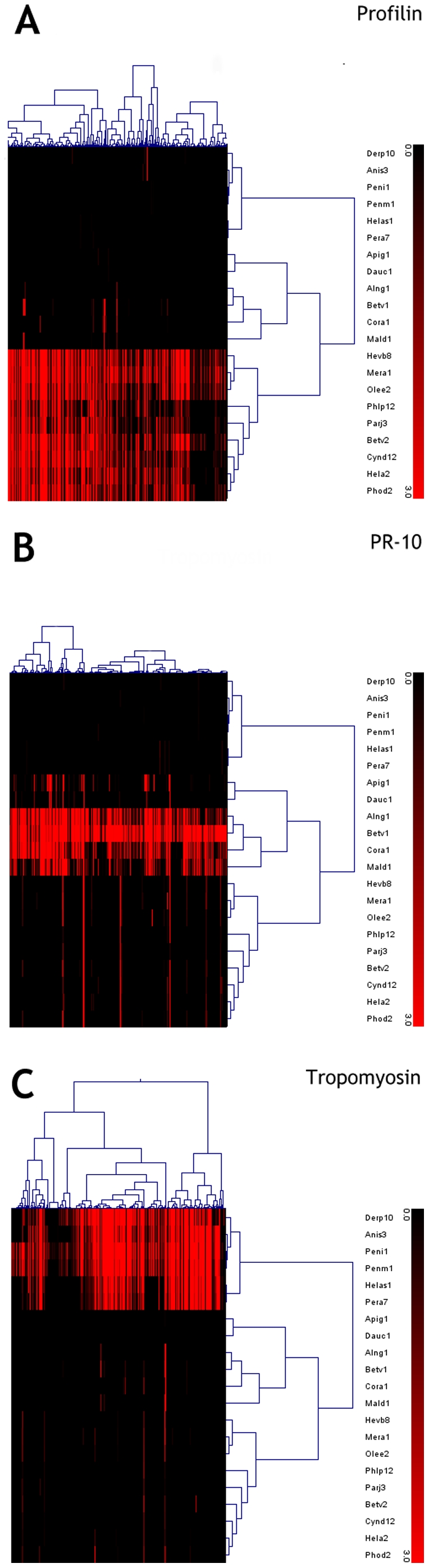
Supervised two-way hierarchical clustering analysis of panallergen IgE values. Subjects had at least one IgE-positive result to the panallergens under study. Allergens are reported on the y-axis, subjects on the x-axis. Black to dark red scale corresponds to IgE values from negative to strongly positive. Further explanations are in the ‘Methods’ section. Panel A: Profilins; Panel B: PR-10; Panel C: Tropomyosins.

Supervised two-way hierarchical clustering analysis, sorting for both allergenic molecules and IgE recognition values, generated distinct clusters of IgE recognition of profilin, PR-10, and tropomyosin molecules, as shown in Figure 4. The IgE recognition of these three distinct groups of molecules was exclusive in 86.4% of cases, as already mentioned, and also the clustering analysis confirmed the high level of exclusivity of IgE recognition of the three panallergens herein evaluated. Plant derived panallergens (PR-10 and profilin molecules) generated a larger cluster of reactivity distinct from the one generated by tropomyosin molecules. In the case of PR-10 molecules, two distinct clusters of IgE reactivity (between Api g 1 and Dau c 1 the first and between Bet v 1, Cor a 1 and Aln g 1 the second) were recorded. Also in the case of tropomyosin IgE reactivity, we observed two distinct clusters of reactivity: one shaped by Hel as 1 and Per a 7, the other by Ani s 3, Der p 10, Pen i 1 and Pen m 1, respectively.

## Discussion

In the present study we perform an in deep analysis of the prevalence of IgE reactivity to panallergens in a large cohort of allergic patients, evaluated by means of a microarray IgE detection system. We studied three groups of molecules belonging to different biological sources and tissues (profilin, PR-10, and tropomyosin), involved at the same time in respiratory and food allergy. Such allergens have been reported as representative examples of IgE “cross reactivity” between otherwise unrelated organisms [19]–[26].

We found that panallergen IgE recognition is exclusive in a large part of our Italian cohort. More than 86% of patients produce specific IgE to a group of panallergens but not to the others. As a result, in the vast majority of cases, the multiple reactivity to inhalant or food allergenic sources was caused by the sensitization to at least one representative molecule of a single panallergen group. Similarly, tropomyosin reactive individuals did not raise an IgE response to other panallergens, as also shown by the hierarchical clustering analysis (Figure 4). Comparatively we showed that when allergenic molecules sensitizing allergic patients by the inhalation route are considered a significant number of concurrent sensitization is recorded. Such number is higher when the exclusive inhalation route of sensitization and the following exposure is considered.

The use of the same approach, originally designed for genomic evaluation, in the epidemiological assessment of IgE reactivity to panallergens, might help in the management of large amount of data generated by serial proteomic evaluation of hundreds of molecules at the same time, in the same group of patients [7]. In this way, it is possible to identify subsets of patients having a similar IgE recognition profile. At the same time, it's also possible to confirm that molecules belonging to a given group of panallergen generate distinct clusters, as a result of the IgE recognition of the same group of molecules, at the same time by the same group of patients. Presumably, all the tropomyosin sensitized patients are also exposed to pollen and food PR-10 and profilins, but the vast majority of the cases did not recognize the other panallergens. If such behavior is more stringent when the IgE reactivity to tropomyosin is compared to the IgE recognition of the plant-derived molecules, the same finding is less obvious when we analyzed the latter groups of panallergens. Given that the possible role of HLA association in the mechanism of IgE response is not fully understood, further investigations of the immune mechanisms at the level of allergen-specific T cells and TCR-MHC/peptide interactions, could identify the MHC restriction elements capable of determine the preferential recognition of a given panallergen group instead of another. In absence of a genetically driven mechanism and in the light of IgE recognition of conformational epitopes, new hypotheses should be made to explain our findings.

Furthermore, as in the case of profilin IgE sensitization, molecules not commonly found in the environment such as Hev b 8, hardly found in latex gloves [27], or Mer a 1, *Mercurialis annua* pollen is rarely found in the atmosphere [28], turned out to be the most frequently recognized by patients' IgE, most likely primarily sensitized by other profilins. Such findings would suggest to carefully considering a molecule as the primary sensitizer merely on the basis of its higher or more frequent IgE reactivity. In the specific cases, latex and *Mercurialis* profilins could act just as the best “profilin” reagents for specific IgE detection among the many profilins already identified. Better conclusions on this issue could be drawn when some profilins, like Cuc m 2, the muskmelon profilin, commonly causing patients' reported symptoms [29], will be available for testing. Such observation can apply also to PR-10, as far as patients in the geographical area under consideration are not exposed to birch pollen [30], whereas IgE recognition of tested tropomyosins does not seem to suggest any leading role in either a molecule as sensitizer or just as the best reagent to detect group-related specific IgE. As in part already reported for the studied panallergens and other allergenic groups [31]–[33], in the case of profilin and PR-10, IgE co-recognition of allergens seems not to be as obvious as it should be from their sequence homology and structure similarity.

Discovering that the production of IgE is preferentially addressed to a panallergen rather than another is relevant also considering the different clinical phenotypes that these molecules could determine. Clinical reactions of panallergens, in fact, might range from very mild symptoms in the case of profilin reactivity [34], [35], to mild or sometimes severe symptoms if PR-10 molecules are involved [36], [37], to often severe reactions when IgE reactivity is directed to other panallergens, such as lipid transfer proteins [38], [39]. The preferential IgE recognition of the panallergens would ease the clinical approach to patients sensitized to one or another group.

Another topic addressed by our study is represented by the possibility to evaluate the IgE reactivity to several representative molecules within each group of panallergens by means of the microarray [40]. In fact, the IgE epitope recognition of homologous molecules, within a given group of panallergens, could be the result of the somatic hyper-mutation and affinity maturation after the initial IgE recognition of the primary sensitizer as determined by the patient's different environmental exposure [41], [42]. As shown in Figure 3, a significant relationship within every single group of molecules was clearly shown, as the consequence of an IgE co-recognition of all the homologous molecules belonging to the given panallergen group [43]. At the same time, different clusters of patients could be identified on the basis of the diversity of IgE recognition within every single group of panallergen (Figure 4). For instance, in the case of PR-10, two distinct subsets of patient could be identified: those IgE reactive to pollen-derived molecules (Bet v 1, Cor a 1 or Aln g 1) and those also reactive to food-derived PR-10 molecules (Dau c 1, Api g 1 or Mal d 1) (Figure 4). Similarly, in the case of tropomyosin reactivity, two distinct subsets of patients could be identified, the first reactive to Hel as 1 and Per a 7, and the other reactive to all the other tropomyosins. As a consequence, the presence of distinct representatives of a given group of panallergen represents an added value of the microarrayed system for the evaluation of IgE reactivity: it gives higher consistency to obtain more reliable results and could add further information on the hypothetical sensitizer in a given group of patients (Figure 3 and 4). All findings reported from using different analytical approaches require further evaluation using wet lab experimental studies using the same microarray-based technique, as already reported for another panallergen group, namely the lipid transfer proteins [44]–[46].

Overall, allergy diagnosis based on allergenic molecules is crucial for a correct evaluation of patients sensitized to panallergens. Molecular analysis represent the only way to establish the presence of new profiles represented by profilin, tropomyosin or PR-10 reactivity, conditions that are addressed to a preferential recognition of one panallergen, in the vast majority of cases, as suggested by our observations.

In conclusion, protein microarray techniques applied in allergy diagnosis allow the identification of several IgE reactivity patterns and possibly could lead to a better knowledge of the relationship between basic immunological mechanisms and clinical symptoms. We would put much emphasis on the opportunity that micro-technologies give us to broad our allergy testing and, at the same time, get detailed and in deep views of unselected patient's IgE immune recognition [7].
